# Using spatially explicit surveillance models to provide confidence in the eradication of an invasive ant

**DOI:** 10.1038/srep34953

**Published:** 2016-10-10

**Authors:** Darren F. Ward, Dean P. Anderson, Mandy C. Barron

**Affiliations:** 1 Landcare Research, PB 92170, Auckland, New Zealand; 2 School of Biological Sciences, University of Auckland, PB 92019, Auckland, New Zealand; 3 Landcare Research, PO Box 69040, Lincoln, New Zealand

## Abstract

Effective detection plays an important role in the surveillance and management of invasive species. Invasive ants are very difficult to eradicate and are prone to imperfect detection because of their small size and cryptic nature. Here we demonstrate the use of spatially explicit surveillance models to estimate the probability that Argentine ants (*Linepithema humile*) have been eradicated from an offshore island site, given their absence across four surveys and three surveillance methods, conducted since ant control was applied. The probability of eradication increased sharply as each survey was conducted. Using all surveys and surveillance methods combined, the overall median probability of eradication of Argentine ants was 0.96. There was a high level of confidence in this result, with a high Credible Interval Value of 0.87. Our results demonstrate the value of spatially explicit surveillance models for the likelihood of eradication of Argentine ants. We argue that such models are vital to give confidence in eradication programs, especially from highly valued conservation areas such as offshore islands.

The detection of low numbers of individuals is a common problem for biological invasions and pest management^1^,^2^. Detectability is important for surveillance at the border, for surveys of spread once a species has established, and for eradication programs.

Ants are recognised globally as significant exotic invaders^3^. However, until recently, relatively little had been published on detectability for invasive ants^4^,^5^,^6^, despite invasive ants being prone to poor detection because of their small size, variable foraging habits, cryptic nature, and strong association with human transportation^3^. One species, the Argentine ant, *Linepithema humile*, is highly invasive and has been accidentally introduced by human trade to many countries throughout the world^7^,^8^,^9^. It has invaded numerous habitats, including coastal sage scrub in southern California, riparian woodland in California, matorral in Chile, fynbos in South Africa, subalpine shrubland in Hawaii, and oak and pine woodland in Portugal^3^. In terms of their impacts on biodiversity, the primary effect of Argentine ants is the displacement of native ant species^3^.

Invasive ants have particular significance for New Zealand, where there are few native ant species^10^, and many globally invasive ant species are not yet present. Argentine ants were first discovered in New Zealand over two decades ago^11^. However, with human-mediated dispersal, they are now relatively widespread, but patchily distributed, in many North Island towns and cities, and also in several locations in the South Island^11^. In New Zealand, Argentine ants are known to occupy a range of open-canopy ecosystems, including native habitats and anthropogenic environments^12^,^13^,^14^. Recent studies have shown Argentine ants can interfere with the success of biological control agents released for the control of boneseed, *Chrysanthemoides monilifera* ssp. *monilifera*^15^ and also increase the reproductive output of this weed^16^. Argentine ants have also been shown to interrupt the decomposition processes via displacement of invertebrate communities^17^.

One of the key goals of Argentine ant management in New Zealand is the eradication, and prevention of re-establishment, of Argentine ant populations from offshore islands that act as conservation sanctuaries for endangered bird^18^. A major part of this goal is developing surveillance and analytical methods to increase confidence that offshore islands are free of Argentine ants, or that a population has been successfully eradicated.

The aim of this paper is to demonstrate how spatially explicit surveillance data can be used to estimate the probability that Argentine ants have been eradicated from a large area. The concept is also applicable to other species of invasive ants, and indeed other invasive taxa.

## Results and Discussion

### Probability of eradication

The probability of eradication increased sharply as each survey was conducted, reflecting the cumulative surveillance effort and system sensitivity with no ants detected (Table 1). The overall median probability of eradication of Argentine ants from all survey and surveillance methods using parameter set 2 was 0.957 (Table 1). There was a high level of confidence in this result, with a high Credible Interval Value of 0.87 (i.e. 87% of the probability of eradication estimates were greater than the threshold value of 0.9). The overall sensitivity of the surveillance system was relatively insensitive to the parameter set used, although parameter set 2, with different decay curves for different search methods, gave greater overall sensitivity (Table 1). In addition, the estimates of the probability of eradication were insensitive to the prior distributions. The parameter set1 with highly informative priors produced a posterior probability of eradication of 0.943, but all other trials were above 0.95. Using an uninformed prior (i.e. a Pert with a range 0–1) that is skewed low (value = 0.25) requires sizable surveillance data in order to achieve a posterior >0.95. Given that our results meet or exceed this target, there is substantial reason to be confident that Argentine ants have been eradicated.

### Eradication of ants

Invasive ants are generally regarded as being very difficult to eradicate^19^,^20^. Eradication is often possible from small scale plots, or when a species is discovered early in its establishment phase in a new area. Larger-scale eradication of invasive ants, whilst possible, remains much more demanding^19^,^21^. Currently one of the major challenges for Argentine ant eradication programmes is finding the location of small ‘survivor’ nests after the initial control operation has occurred. Such nests remain restricted to a very small area (<few metres), and thus a large proportion of time and resources are spent on finding and controlling only 1% of ants.

Recent work on formulating requirements for the eradication success of invasive ants includes a number of factors, such as the importance of operational requirements (e.g. lines of authority, sufficient resources for the task) and on early intervention^19^, which currently gives a far greater chance of success for invertebrate eradication efforts^20^. However, improving detectability at low densities is also one of the most critical factors to increase the success of ant eradication^19^. Typically ‘eradication success’ is declared after a certain time when the pest has not been found, for example, two years post-treatment has been the minimum standard used for most published ant^22^ and vertebrate eradications^23^. However, the lack of detection after just waiting a certain time period does not prove a pest is absent. A pest may still be present in an area somewhere but remains undetected through poor surveillance coverage and/or intensity, or purely by chance. That is why it is critical to interpret negative surveillance data (non-detections) in light of the surveillance system sensitivity.

### Proof of eradication modelling

Modelling the probability of eradication is a valuable technique, providing a level of confidence in the overall result and justification for the allocation of resources required to meet a set probability level. This type of modelling is also very useful in understanding the resources needed to achieve certain confidence levels. For example, modelling will help avoid prematurely declaring success due to insufficient survey effort or, conversely, avoid wasting resources on surveys when the pest has been eradicated from an area. Modelling may also identify spatial gaps in surveys, where search effort has been too low, allowing for subsequent surveys to be directed to search these areas. This ensures there are no refuges available for a pest population, and ultimately gives increased confidence of the overall probability of eradication.

## Conclusions

Here, we demonstrated a technique that estimated the confidence in the eradication of an invasive ant based on defining the relationship between surveillance effort and the probability of detecting the target organism, a technique previously used in eradications of vertebrates^23^ and wildlife disease^24^. In general, this modelling technique provides useful information about the probability of eradication of a pest species, and thus gives confidence of eradication at very low densities. Such modelling could be applied to many populations of Argentine ants, and assist in the eradication of this pest from many locations around the world.

## Methods

### Study site

The study took place on Schoolhouse Bay (−36.428°S, 174.831°E), one of many bays on Kawau Island, a large (19 km^2^) island in the Hauraki Gulf, <2 km from the mainland and about 40 km north of Auckland, New Zealand. Argentine ants were first found at Schoolhouse Bay in 2010. Kawau Island has a warm temperate climate and the majority of the island is covered in scrub and regenerating forest. Kawau Island is part of the Hauraki Gulf Marine Park, an extensive area of 50 islands spread over 1.2 million hectares that includes publically accessible recreation areas and sanctuaries for endangered species. Several islands are subject to the removal of invasive animals, including Argentine ants.

### Ant control and post-control surveys

Control of Argentine ants at Schoolhouse Bay took place on 3 October 2012 with a team of 12 people. Xstinguish™ Argentine ant bait was laid across the infested area and a 50 m buffer zone (~3 ha in total) at ~2 m intervals. Four post-control surveys were undertaken (12th March 2013, 29th October 2013, 12th November 2013, and 21st February 2014) to monitor the initial outcome of the control operation, and quantify the probability of successful eradication. Three different surveillance methods were utilised to detect Argentine ants during these surveys: (i) visual hand searching (visual searching in and around vegetation and houses, lifting logs and stones); (ii) baited vials (n = 500 vials, with a non-toxic food source [paste of mixed sausage meat and sugar] placed into a plastic vial, opening with diameter of 25 mm); and (iii) a sniffer-dog (trained to detect the presence of Argentine ants^25^).

**Figure 1:**
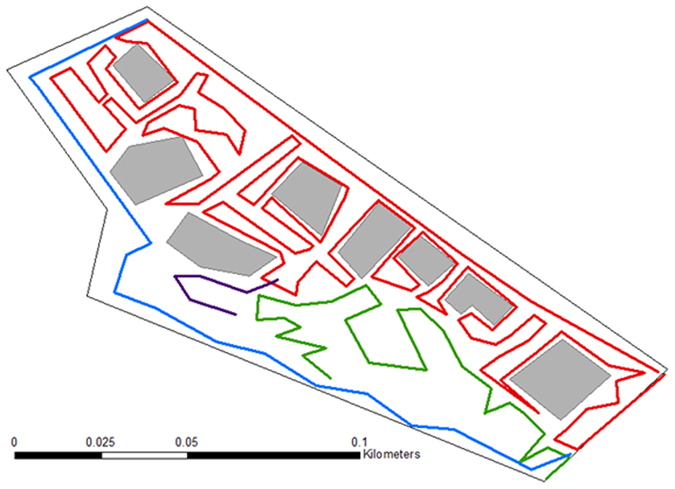
Schematic map of Schoolhouse Bay study site and location of paths where searching for Argentine ants was conducted (based on GPS locations). Grey shaded areas are houses. Path 1 (red), Path 2 (green), Path 3 (purple), Path 4 (blue).

We used several ‘paths’ to cover the entire Schoolhouse bay area. These paths were recorded with GPS and used for all three surveillance methods (Table 2; Fig. 1). Areas covered by houses were excluded from the area of inference because no internal searching of houses has been carried out by us (but informally by residents). Data was analysed with a spatially explicit surveillance model, as described below. The model estimated the sampling sensitivity for each survey and updated the overall probability of eradication with each survey.

### Proof of eradication modeling

We used a spatially explicit surveillance model of Anderson *et al*.^24^, initially developed for disease eradication in vertebrates, to estimate the probability that Argentine ants had been eradicated from the Schoolhouse Bay area given negative surveillance results since control was applied. The model estimated the overall sensitivity of the surveillance (i.e. the probability of detecting Argentine ants given they were present) using a 1-m grid-cell resolution across the modelled landscape. Following a survey where ants were not detected, the sensitivity of the surveillance method was used to update the probability of ant eradication using Bayes theorem.

Under the framework from the aforementioned model, there are two key parameters for estimating the probability of detection in a given grid cell^24^. The first was the maximum probability of detecting an ant or its nest (*g*_*0*_) if the surveillance device was placed directly on top of it. The second was a spatial decay parameter (*σ*), which modifies how the probability of detection declined with distance from the surveillance device. Consequently, a device can detect ants or nests that were in the same or neighboring grid cells.

**Figure 2:**
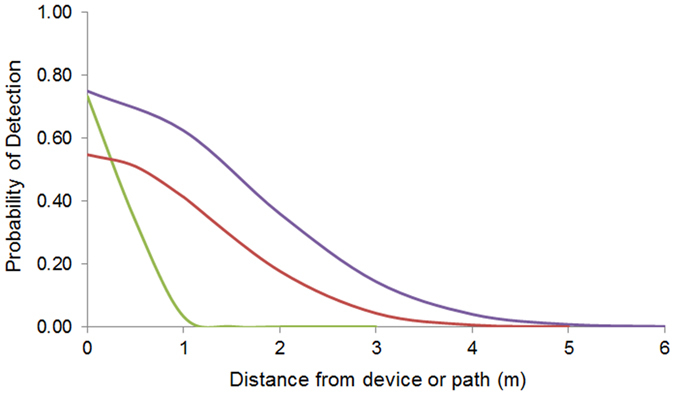
Half-normal function describing the probability of detecting an Argentine ant or nest with distance from a device (baited vials) or from a point along a path (person visual, sniffer dog). Visual searching (green), sniffer dog (purple), baited vials (red).

The baited vials were spaced 2 m apart along the path. The *g*_*0*_ and *σ* parameters for the baited vials were derived by fitting a half-normal function to the curve shown in Fig. 2 of Stringer *et al*.^6^, which described the probability of detecting foragers from a small nest of the fire ant (*Solenopsis invicta*) using baited vials placed out for 1–2 hours at different distances from the nest. The fitted values were: *g*_*0*_ = 0.548 and *σ* = 1.331 (Fig. 2). Although the above parameters were developed for fire ants, they are biologically relevant to Argentine ants, as when ant colonies (no matter what species) are very small, they have a very small foraging area, and thus their detection (by baits, visual searching, etc.) is harder.

We developed two model sets. Because human visual searchers and sniffer dogs move continuously along a path, we divided the path up into regularly spaced ‘search points’. In the first instance (parameter set1) we assumed the same *g*_*0*_ and *σ* values as those described by Stringer *et al*.^6^ and 2-m spacing between search points for all three surveillance methods. Secondly (parameter set2), we attempted to better represent the detection range of visual searching and sniffer dogs, for which information was specifically available for Argentine ants^25^,^26^. We modified values from Stringer *et al*.^6^, restricting visual searching to a 1-m decay and increasing the decay of the sniffer dog to 4 m. We also changed the search-point spacing to give an equal detection probability along the path bearing (distance = 0) as a result of the overlapping detection kernels. For the visual searchers in parameter set2 we used *g*_*0*_ = 0.733, *σ* = 0.4 and a search-point spacing of 1 m. For the sniffer dogs in parameter set2 we used *g*_*0*_ = 0.750, *σ* = 1.65 and a search-point spacing of 2 m (Fig. 2). This was equivalent to a per-cell probability of detection 0.75 and 0.9 of detecting an ant nest if the search point of person or dog, respectively, was in the same cell as the ant. In both parameter sets *σ* and *g*_*0*_ parameter values were allocated a standard deviation equivalent to a 10% Coefficient of Variation around the mean value to account for the uncertainty in model parameters.

A relatively pessimistic ‘prior’ probability of eradication being successful was specified using a Pert distribution with a most likely value of 0.25 (range 0–0.75). The ‘prior’ is our estimated probability that ants were absent from the island at the time of the first post-treatment surveys. We suggest that the value of 0.25 is appropriate given the uncertainty in the control operation. We assessed the sensitivity of the estimated probability of eradication to the prior distribution. Retaining a most likely prior value of 0.25, analysis for both parameter sets were repeated using a highly informative distribution (range 0–0.4) and an uninformative distribution (0–1). The design prevalence or the minimum number of 1-m^2^ grid cells within the Schoolhouse Bay Area likely to be infested if ants were still present was set to one (i.e. one surviving colony).

The annual probability of re-introduction of ants into Schoolhouse Bay was assumed to be very low (because of ongoing education of the public and surveillance in the area), and described by a Pert distribution with a most likely value of 0.01 (range 0–0.012). Point locations of the baited vials used in this study were derived by spacing 500 vials along the Schoolhouse Bay search paths^27^ using the ArcGIS 10.2.1 ‘Construct Points’ function. The same tool was used to assign equally spaced search points for the visual searchers and sniffer dog.

## Additional Information

**How to cite this article**: Ward, D. F. *et al*. Using spatially explicit surveillance models to provide confidence in the eradication of an invasive ant. *Sci. Rep*. **6**, 34953; doi: 10.1038/srep34953 (2016).

## Figures and Tables

**Table 1:** Model results for the median system sensitivity and probability of eradication with 90% credible intervals in brackets.

Parameter set	Survey Date	Median system sensitivity	Probability of eradication	CIV
1	March 2013	0.448 (0.443, 0.453)	0.419 (0.112, 0.690)	0
	October 2013	0.644 (0.640, 0.647)	0.668 (0.259, 0.860)	0.001
	November 2013	0.682 (0.679, 0.686)	0.863 (0.523, 0.951)	0.309
	February 2014	0.682 (0.679, 0.685)	0.951 (0.773, 0.983)	0.831
2	March 2013	0.149 (0.147, 0.150)	0.312 (0.074, 0.607)	0
	October 2013	0.713 (0.709, 0.718)	0.611 (0.215, 0.843)	0
	November 2013	0.734 (0.730, 0.739)	0.855 (0.508, 0.952)	0.293
	February 2014	0.736 (0.732, 0.741)	0.957 (0.797, 0.986)	0.870

The credible interval value (CIV) is the proportion of the posterior probability of eradication that is greater than 0.9 (CIV threshold). Parameter set 1: detection and decay parameters equal for all three surveillance methods. Parameter set 2: detection and decay parameters individualised for each surveillance method.

**Table 2:** Details of the surveillance methods utilised along different paths (see Fig. 2) and survey periods.

Path	Surveillance Method	March 2013	October 2013	November 2013	February 2014
1	Baited vial		×	×	×
2	Baited vial		×	×	×
3	Baited vial			×	×
4	Baited vial			×	
1	Visual	×	×	×	×
2	Visual	×	×	×	×
3	Visual	×	×	×	×
4	Visual	×		×	×
1	Dog		×	×	×
2	Dog		×	×	×
3	Dog				×
4	Dog		×	×	×

## References

[b1] Venette R. C., Moon R. D. & Hutchison W. D. Strategies and statistics of sampling for rare individuals. Annu. Rev. Entomol. 47, 143–174 (2002). 11729072 10.1146/annurev.ento.47.091201.145147

[b2] Regan T. J. . Optimal eradication: when to stop looking for an invasive plant. Ecol. Lett. 9, 759–766 (2006). 16796564 10.1111/j.1461-0248.2006.00920.x

[b3] Holway D. A., Lach L., Suarez A. V., Tsutsui N. D. & Case T. J. The causes and consequences of ant invasions. Annu. Rev. Ecol. Syst. 33, 181–233 (2002).

[b4] Stanley M. C. . Optimising pitfall sampling for the detection of Argentine ants, *Linepithema humile* (Hymenoptera: Formicidae). Sociobiology. 51, 461–472 (2008).

[b5] Schmidt D. . Finding needles (or ants) in haystacks: predicting locations of invasive organisms to inform eradication and containment. Ecol. Appl. 20, 1217–1227 (2010). 20666245 10.1890/09-0838.1

[b6] Stringer L. D. . Sampling efficacy for the red imported fire ant *Solenopsis invicta* (Hymenoptera: Formicidae). Environ. Entomol. 40, 1276–1284 (2011). 22251738 10.1603/EN11002

[b7] Suarez A. V., Holway D. A. & Case T. J. Patterns of spread in biological invasions dominated by long-distance jump dispersal: insights from Argentine ants. PNAS. 98, 1095–1100 (2001). 11158600 10.1073/pnas.98.3.1095 PMC14714

[b8] Silverman J. & Brightwell R. J. The Argentine ant: challenges in managing an invasive unicolonial pest. Ann. Rev. Entomol. 53, 231–252 (2008). 17877449 10.1146/annurev.ento.53.103106.093450

[b9] Roura-Pascual N. . The relative roles of climatic suitability and anthropogenic influence in determining the pattern of spread in a global invader. PNAS. 108, 220–225 (2011). 21173219 10.1073/pnas.1011723108 PMC3017164

[b10] Ward D. F. The diversity, community composition and seasonality of native ants in northern New Zealand. Myrmecol. News. 12, 195–200 (2009).

[b11] Ward D. F. . Twenty years of Argentine ants in New Zealand: past research and future priorities for applied management. N. Z. Entomol. 33, 67–78 (2010).

[b12] Harris R., Ward D. F. & Sutherland M. A. Assessment of the risk of Argentine ant, *Linepithema humile*, to natural environments in New Zealand. Landcare Research Report. 1–33 (2002).

[b13] Lester P. J., Baring C. W., Longson C. G. & Hartley S. Argentine and other ants (Hymenoptera: Formicidae) in New Zealand horticultural ecosystems: distribution, hemipteran hosts, and review. N. Z. Entomol. 26, 79–90 (2003).

[b14] Stringer L. D., Stephens A. E., Suckling D. M. & Charles J. G. Ant dominance in urban areas. Urban Ecosys. 12, 503–514 (2009).

[b15] Paynter Q. . Biotic resistance: facilitation between invasive Homoptera and invasive ants limits the establishment of an introduced weed biocontrol agent in New Zealand. Biocontrol. 63, 188–194 (2012).

[b16] Stanley M. C. . Invasive interactions: Can Argentine ants indirectly increase the reproductive output of a weed? Arthropod-Plant Inte. 7, 59–67 (2013).

[b17] Stanley M. C. & Ward D. F. Impacts of Argentine ants on invertebrate communities: below-ground consequences? Biodivers. Conserv. 21, 2653–2669 (2012).

[b18] Jay M. Recent changes to conservation of New Zealand’s native biodiversity. New. Zeal. Geogr. 61, 131–138 (2005).

[b19] Hoffmann B. D. Eradication of populations of an invasive ant in northern Australia: successes, failures and lessons for management. Biodivers. Conserv. 20, 3267–3278 (2011).

[b20] Tobin P. C. . Determinants of successful arthropod eradication programs. Biol. Invasions. 16, 401–414 (2014).

[b21] Hoffmann B. D. & O’Connor S. Eradication of two exotic ants from Kakadu National Park. Ecol. Manage. Res. 5, 98–105 (2004).

[b22] Hoffmann B. D. Ecological restoration following the local eradication of an invasive ant in northern Australia. Biol. Invasions. 12, 959–969 (2010).

[b23] Ramsey D. S. L., Parkes J. P. & Morrison S. A. Quantifying eradication success: the removal of feral pigs from Santa Cruz Island, California. Conserv. Biol. 23, 449–459 (2009). 19040652 10.1111/j.1523-1739.2008.01119.x

[b24] Anderson D. P. . A novel approach to assess the probability of disease eradication from a wild-animal reservoir host. Epidemiol. Infect. 141, 1509–1521 (2013). 23339965 10.1017/S095026881200310X PMC9151828

[b25] Ward D. F. . A ‘sniffer’ dog to detect Argentine ants. *Protect*. Spring, 15–16. (2014) Accessible: http://biosecurity.org.nz/images/pdfs/Protect/Protect-Spring-2014.pdf (Date of access: 01/10/2014).

[b26] Ward D. F. & Stanley M. C. Site occupancy and detection probability of Argentine ant populations. J. Appl. Entomol. 137, 197–203 (2013).

[b27] Ward D. F., Anderson D. & Barron M. B. Progress on the eradication of Argentine Ants. Envirolink Advice grant 1471-NLRC172. Landcare Research report LC1876. p27. (2014). Accessible: http://www.envirolink.govt.nz/envirolink-reports/ (Date of access: 24/6/2016).

